# Some Points in the Toilet of the Peritoneum1Read at the thirty-third annual meeting of the Medical Association of Central New York, held at Rochester, N.Y., October 16, 1900.

**Published:** 1901-01

**Authors:** Charles E. Congdon

**Affiliations:** Buffalo, N. Y.


### SOME POINTS IN THE TOILET OF THE PERITONEUM.[1]

BY CHARLES E. CONGDON, M. D., Buffalo, N. Y.

IN LISTENING to papers and discussions, I observe that many abdominal surgeons conclude their report of their cases as follows: After thoroughly washing or flushing the abdomen, proper drains inserted or the abdomen closed, until it has become a habit with some men to flush the abdomen for all sorts of reasons and for no reason at all.

1. Read at the thirty-third annual meeting of the Medical Association of Central New York, held at Rochester, N.Y., October 16, 1900.

The excuse given by some operators is to get rid of disorganised blood-clots; others to irrigate out the filth after every operation. Some believe there is an advantage in irrigation in pus formation; still others report cases of general septic peritonitis cured by irrigation or flushing the general peritoneal cavity. A few even eviscerate in order to more thoroughly execute this procedure.

Tait was a thorough believer in abdominal irrigation; he used, however, common tap water. I am inclined to think that today, perhaps, the large majority of operators are followers of Tait, with the exception that they use a normal sterile saline solution.

The advantages that are claimed for this procedure are in the direct line of cleanliness, hemostasis and stimulation; the disadvantages are a greater liability to infection from imperfectly sterilised solution or contamination in the manipulation, the amount of time consumed requiring a longer anesthesia, the liability to disseminate infection, the injury to the peritoneum from the solution being too hot or too cold, mechanical irritation from greater manipulation, and last, but not least, the absolute impossibility to render the peritoneum aseptic by washing, even though you eviscerate

Some surgeons are so wedded to irrigation that they honestly believe that their patients would succumb were they not thoroughly irrigated. I am frank to confess that I was one of this class, as I have even eviscerated in order to carry out this procedure in a more thorough manner. However, for the last year and a half I have not irrigated, scrubbed or flushed the peritoneal sac in part or in whole.

The following table will show the greater number of pus cases I have had during the last eighteen months: Twenty-two cases of tubo-ovarian abscess with pus in the pelvis; ten cases of appendicitis with ruptured appendix with pus in the pelvis, flank or both, six of them apparent or so-called general peritonitis; one case of cholecystitis, the abdomen full of sero-floculent material; two cases of ectopic gestation, the abdomen filled with blood-clots and greatly distended; one case of perforating ulcer of the stomach, with a collection of two ounces of pus; in this case there was resection of a portion of the stomach, the gall-bladder and appendix were in a mass of adhesions, a large stone was removed from the gall-bladder, the incision sutured, gall-bladder dropped and appendix removed.

In this series only one death has occurred, and that was in a case of appendicitis; the patient was moribund when placed on the table and should not have been operated upon. While I could readily bring elaborate reports of these cases, yet I do not wish to bore you.

The method which I have usually followed, is to make a small incision; if localised inflammatory condition exists, to wall it off with sterilised gauze, then thoroughly remove the pathologic condition present, leaving no stump or pedicle; absorb all purulent material with gauze, rubbing or irritating the peritoneum as little as possible, not even removing the plastic exudate, believing and trusting that the peritoneum in the immediate vicinity of the inflammatory process is immune and better capable of caring for itself when uninjured; being a firm believer in the bacteriologic causes of disease and remembering laboratory experiments, how feeble and nonvirulent pathogenic organisms develop, if developed at all, on old culture media, and yet how virulent they become if transferred to a different or fertile media.

This certainly answers one of the questions as to why we should disturb as little as possible tissues surrounding a localised abscess; I care not whether it be in the peritoneum or other organs or tissues of the body. How many of us have seen a pelvis full of pus, the patient so exhausted from the virulence of the infection and prolonged illness, that it seemed as though there was no chance, and yet a quick incision above or below, as seemed most suitable, not taking time for irrigation, for fear the patient would succumb on the table, a rapidly inserted drain, and find the patient on the following day nourishing well and inquiring when she could go home.

We occasionally meet with noncircumscribed collections of purulent material in the abdomen. The only reason that I can ascribe for its not becoming rapidly diffuse is that nature renders the peritoneum an unsuitable culture medium, or the nonvirulence of the infection, or both. These cases, although with a high temperature and rapid pulse, all yield by the complete removal of the exciting cause; small drains, no attempt to close the incision, that being left for a secondary operation. In some of these cases the entire abdomen appears to be filled with pus; the temptation to eviscerate and flush is very great.

These cases have readily yielded in my hands to the same treatment as in the last condition stated; formerly under the irrigation treatment many succumbed. I do not believe this to be a true general infection, but simply a puriform collection. In the true form of general peritonitis the prognosis is exceedingly grave, for patients rarely survive such an infection.

Injections of pure cultures of purulent material directly into the peritoneal cavity without injury to the peritoneum cause only temporary if any reaction, showing the wonderful power of the peritoneum to care for itself, and yet the uniformly fatal results, if combined with trauma, has led me to become perhaps too enthusiastic in the nonuse of irrigation. However this may be, I am inclined to believe that many more lives could be saved by not too radical but by more simple surgical procedures.

1034 JEFFERSON STREET.

